# Handheld UV-C Fluorescence Imaging with Cascaded Denoising and Deep Learning for *Escherichia coli* Concentration Classification on Diverse Food-Contact Surfaces

**DOI:** 10.3390/s26216699

**Published:** 2026-10-23

**Authors:** Zafar Iqbal, Thomas F. Burks, Sriram Thota, Akash Singh, Snehit Vaddi, Pappu Yadav, Jianwei Qin, Moon S. Kim, Mark A. Ritenour, Jiuxu Zhang, Fartash Vasefi

**Affiliations:** 1 Department of Agricultural and Biological Engineering, University of Florida, Gainesville, FL 32611, USA; 2 Department of Computer & Information Science & Engineering, University of Florida, Gainesville, FL 32611, USA; 3 Department of Agricultural and Biosystems Engineering, South Dakota State University, Brookings, SD 57007, USA; 4 USDA/ARS Environmental Microbial and Food Safety Laboratory, Beltsville Agricultural Research Center, Beltsville, MD 20705, USA; 5 Department of Horticultural Sciences, University of Florida, Fort Pierce, FL 34945, USA; 6 SafetySpect Inc., Grand Forks, ND 58202, USA

**Keywords:** *E. coli*, food safety, fluorescence imaging, CSI-D+, deep learning, denoising, cascaded denoising, food-contact surfaces, XAI

## Abstract

Rapid, non-destructive assessment of *Escherichia coli* contamination levels on food-contact surfaces is important for food safety monitoring and sanitation verification. This study developed a deep-learning framework integrating UV-C fluorescence imaging and cascaded denoising to classify predefined *E. coli* concentration levels on four food-contact surfaces. A SafetySpect CSI-D+ handheld system acquired fluorescence images at four concentration levels: Control (0), 10^6^, 10^7^, and 10^8^ CFU/mL, on stainless steel, white rubber, white plastic, and black plastic. Five image-denoising operators (wavelet thresholding, NLM, unsharp masking, Wiener filtering, and RL-TV) were evaluated in sequential and parallel configurations. Eight deep-learning classifiers, including five YOLO11-cls variants, EfficientNetB7, ConvNeXtBase, and ViT, were benchmarked using consistent evaluation protocols. Classification performance varied with surface type and denoising strategy. The sequential Wavelet → Wiener → RL-TV cascade achieved the highest observed mean test accuracy (94.18%), with surface-specific accuracies of 97.57%, 97.57%, 94.44%, and 87.15% for stainless steel, white rubber, black plastic, and white plastic, respectively. On the most challenging surface (white plastic), the cascade improved accuracy by 11.46 percentage points over the best single operator. YOLO11-based models generally outperformed ViT, although the highest-performing architecture varied by surface. Most misclassifications occurred primarily between 10^6^ and 10^7^ CFU/mL, while the 10^8^ CFU/mL class was consistently classified accurately. Overall, the ordered cascaded denoising can improve robust *E. coli* concentration classification across diverse food-contact surfaces, providing a promising foundation for rapid, non-destructive food safety inspection. The results also highlight the importance of surface-specific preprocessing and deep learning architectures for robust performance across diverse materials.

## 1. Introduction

Foodborne illnesses linked to pathogenic *Escherichia coli* remain a major public health concern and impose a substantial economic burden worldwide [1]. In the United States alone, foodborne illness is estimated to cost roughly $74.7 billion annually, with severe, pathogen-attributable cases accounting for a disproportionate share of that total [2]. Shiga toxin-producing *E. coli* (STEC), particularly serotype O157:H7, is among the most consequential of these pathogens, since infection can progress from severe gastrointestinal illness to hemolytic uremic syndrome and life-threatening kidney failure [3,4]. The continued frequency of high-profile outbreaks underscores how readily contamination propagates through processing and food-handling environments. Recent examples include a 2024 multistate outbreak traced to slivered onions served at a national fast-food chain, which sickened at least 104 people and caused one death [3], and a 2024 outbreak linked to organic walnuts, the first U.S. foodborne STEC outbreak attributed to tree nuts [5]. As a recognized indicator of fecal contamination, *E. coli* can adhere to and persist on food-contact surfaces, facilitating cross-contamination of ready-to-eat products.

Critically, the food-contact surfaces themselves act as active reservoirs rather than passive substrates. In food-processing environments, bacteria readily attach to stainless steel, plastics, and rubber and develop biofilms that are markedly more resistant to sanitizers than free-floating cells, enabling persistent, recurring contamination that routine cleaning and sanitation programs may fail to eliminate [6,7]. These materials differ substantially in texture, porosity, and optical properties, and such differences influence both bacterial survival and the ease with which contamination can be detected optically [6]. Rapid, accurate, on-site quantification of *E. coli* cell concentration across these diverse materials is therefore vital for effective hazard analysis and critical control point (HACCP) programs, sanitation verification, and preventive risk management.

Conventional laboratory methods for *E. coli* detection, including culture-based plating, enzyme-linked immunosorbent assay (ELISA), and polymerase chain reaction (PCR), offer high sensitivity and specificity but differ in speed. Culture typically requires 18 to 48 h, whereas ELISA and PCR return results within a few hours [8,9]. All of these techniques require specialized equipment and trained personnel, limiting their suitability for real-time monitoring in dynamic industrial settings [10,11]. Emerging rapid alternatives have narrowed the speed gap. Deep-learning-enhanced microfluidic fluorescence biosensors have achieved detection limits as low as about 2 CFU/mL within roughly 1.5 h [12], portable electrochemical impedance biosensors now target O157:H7 in complex food matrices [13], and hyperspectral imaging paired with deep neural networks has enabled high-throughput, non-destructive pathogen classification [14]. However, these approaches generally depend on sample collection and preparation, bulky or fragile optics, or benchtop instrumentation, which restricts their use for direct, on-site screening of contamination on surfaces [13]. This leaves a clear need for a reagent-free method that quantifies contamination directly on the surface, in real time, across the varied materials found on a processing line.

Optical technologies based on intrinsic fluorescence offer exactly this kind of non-destructive, reagent-free screening. When excited by ultraviolet-C (UV-C) light near 275 nm, aromatic amino acids (tryptophan and tyrosine) and cellular cofactors in microbial cells emit characteristic autofluorescence signals [15]. These emissions create spatially varying patterns that reflect the presence and relative concentration of biological contaminants on surfaces. The CSI-D+ handheld device from SafetySpect Inc. advances fluorescence-based inspection by combining high-intensity UV-C light-emitting diodes (centered at 275 nm) for simultaneous excitation and disinfection, a sensitive fluorescence imaging sensor, and laser-point and LiDAR-assisted distance control to ensure uniform illumination [16]. This portable instrument captures fluorescence images directly on target surfaces and has shown effectiveness in visualizing organic residues and microbial loads while providing concurrent sanitization [15].

Raw UV-C fluorescence images are nevertheless degraded by several physically distinct processes at once, which shapes the choice of preprocessing. Low-photon fluorescence acquisition is dominated by mixed Poisson–Gaussian noise, and the raw frames additionally contain uneven illumination, material-dependent autofluorescence background, and optical blur from the point-spread function [17]. Because each classical filter is derived under a single noise or blur model, no one method removes all of these degradations simultaneously. Wavelet (discrete wavelet transform) thresholding decomposes an image into multiresolution sub-bands and shrinks high-frequency coefficients, suppressing additive sensor noise while preserving edges [18]. Non-local means (NLM) exploits patch self-similarity, averaging structurally similar regions to remove random noise while retaining fine spatial detail [19]. Wiener filtering applies a frequency-domain minimum-mean-square-error criterion that balances noise suppression against signal fidelity using the estimated spectral signal-to-noise ratio [20]. Unsharp masking, by contrast, is an edge-enhancement operator that heightens the visibility of fluorescence hotspots by subtracting a blurred version of the image, at the cost of amplifying high-frequency content [21]. Richardson–Lucy deconvolution iteratively inverts point-spread-function blur under a Poisson forward model, but it is known to amplify noise and converge poorly as iterations progress, so it is commonly paired with total-variation regularization (RL-TV) to constrain the ill-posed inversion and suppress ringing [22,23]. Because these operators are complementary, combining them generally outperforms any single method. The widely used BM3D algorithm, for instance, unifies non-local averaging, transform-domain thresholding, and Wiener filtering in one pipeline [24], and hybrid wavelet and Wiener deconvolution chains are well established in fluorescence and microscopy restoration [25]. These complementary capabilities motivate a cascaded preprocessing strategy in which sequential operators progressively suppress noise and compensate for residual blur, with the ultimate goal of recovering fluorescence patterns that are more informative for downstream classification.

The quality of this restoration is particularly important because the resulting fluorescence patterns form the basis for concentration classification. Convolutional architectures from the YOLO (You Only Look Once) series excel at real-time detection and multi-class classification on challenging imaging data [26]. The YOLOv11 architecture incorporates refined backbone and neck components that improve feature extraction, inference speed, and accuracy, making it well suited to edge deployment on handheld systems. Compound-scaled convolutional networks such as EfficientNet and modernized designs such as ConvNeXt provide strong benchmarks for comparison [27,28]. More recently, Vision Transformers (ViTs) have introduced a fundamentally different paradigm. Rather than relying on local convolutional receptive fields, a ViT partitions an image into a sequence of patches and applies multi-head self-attention to model long-range, global dependencies across the entire image [29,30]. This global receptive field is complementary to the local inductive bias of convolutional networks, and ViTs have been shown to match or surpass CNNs on image classification when pre-trained on large datasets and transferred to smaller, domain-specific problems [31]. Transformer-based models are now widely adopted in agricultural and food-safety imaging for tasks such as disease classification and crop monitoring [31,32], and wavelet-enhanced transformer denoisers have improved fluorescence-microscopy image quality specifically [25]. Because attention-based and convolutional models capture spatial fluorescence structure differently, benchmarking ViT alongside the YOLO11, EfficientNet, and ConvNeXt families enables a fair comparison of both paradigms. Prior CSI-D+ studies establish the feasibility of this overall approach. Yadav et al. (2024) demonstrated portable concentration detection on citrus leaves and peels [33]; Burks et al. (2025) reached overall accuracies up to 92.6% on citrus leaves with customized YOLOv11 architectures [34]; Vaddi et al. (2025) benchmarked YOLOv11 variants against EfficientNetB7 and ConvNeXtBase on citrus and spinach leaves [35]; and Iqbal et al. (2026) extended the method to stainless steel and white rubber, reaching perfect peak accuracies with wavelet-based denoising [4].

Collectively, these studies demonstrate the effectiveness of CSI-D+ UV-C fluorescence imaging paired with deep learning, with consistent benefits from denoising and explainable AI. However, prior work has largely focused on botanical surfaces or a limited set of materials evaluated in isolation and has relied predominantly on single-stage denoising. It has also not systematically compared convolutional and attention-based classifiers or examined how surface reflectance and background fluorescence influence preprocessing across optically distinct inorganic materials. Following this demonstrated effectiveness, the objective of this study was to develop and validate a framework for the multi-class classification of *E. coli* cell concentration levels (control, 10^6^, 10^7^, and 10^8^ CFU/mL) on four major food-contact surfaces: stainless steel, white rubber, white plastic, and black plastic, using UV-C fluorescence images acquired with the CSI-D+ handheld device. The specific objectives were to: (1) design and evaluate a set of cascaded denoising pipelines built from Wavelet, NLM, unsharp, RL-TV, and Wiener filtering, and justify their selection and ordering; (2) benchmark eight deep-learning classifiers spanning the YOLO11-cls family (n, s, m, l, x), EfficientNetB7, ConvNeXtBase, and a ViT; (3) characterize how surface reflectance and background fluorescence affect denoising behavior and classification performance across the four materials; and (4) apply Eigen-CAM visualization to interpret the discriminative fluorescence features underlying each model’s predictions.

## 2. Materials and Methods

**Figure 1:**
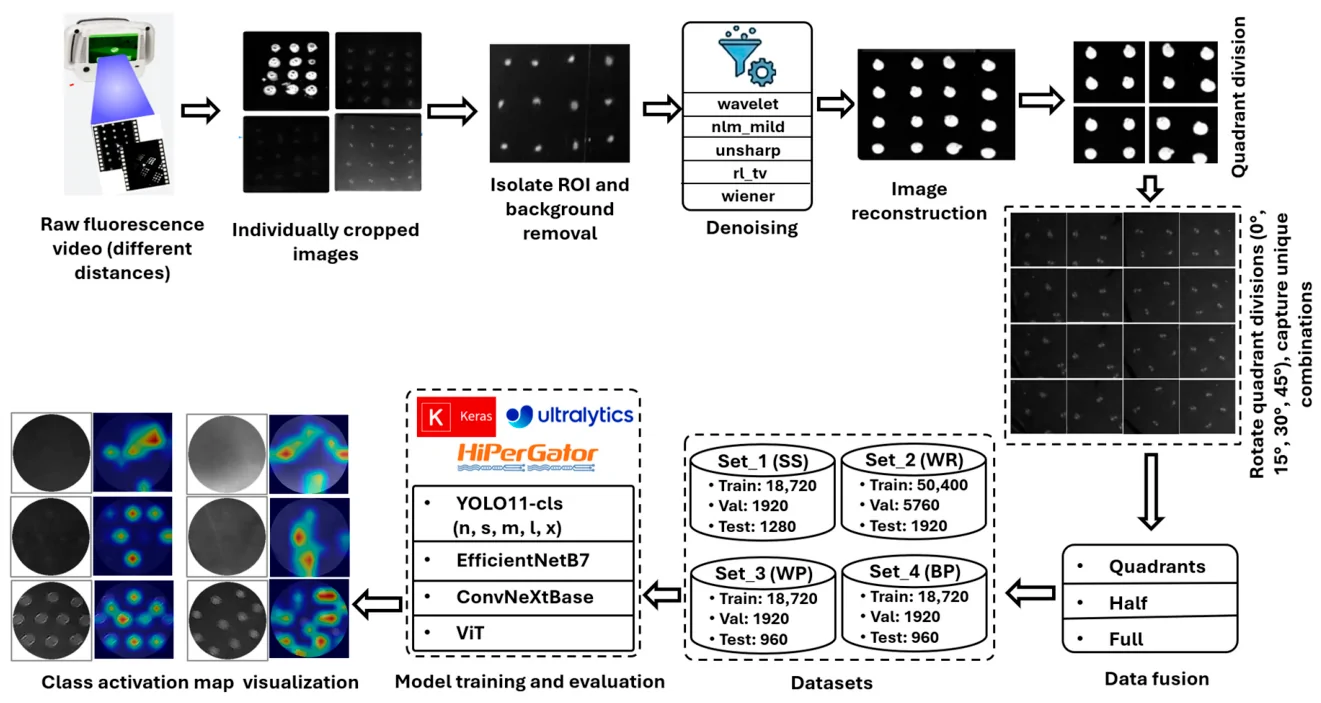
End-to-end pipeline for *E. coli* concentration-level detection on different food processing surfaces using CSI-D+ UV-C fluorescence imaging and deep learning.

The overall workflow for AI-based detection of *E. coli* cell concentration levels on diverse food-contact surfaces is illustrated in Figure 1. It comprises six sequential stages: sample preparation and inoculation; fluorescence image acquisition using the CSI-D+ handheld device; ROI isolation with background removal; cascaded image denoising; image reconstruction with quadrant and multi-scale division; and deep-learning model training and evaluation with Eigen-CAM visualization.

### 2.1. Sample Preparation and Fluorescence Image Acquisition

In this study, a non-pathogenic, generic *E. coli* strain (ATCC 35218) was used. Bacterial cultures were prepared and serially diluted to obtain four target concentration levels: 0 (control), 10^6^, 10^7^, and 10^8^ CFU/mL. The concentrations were verified by colony counting on tryptic soy agar following incubation at 35 °C for 24 h. Samples were inoculated onto four common food-contact surfaces, including stainless steel, white rubber, white plastic, and black plastic, and were allowed to equilibrate under controlled environmental conditions before imaging.

Fluorescence videos were acquired using the second-generation CSI-D+ handheld device (SafetySpect Inc., Grand Forks, ND, USA), equipped with UV-C (275 nm) excitation, co-registered fluorescence imaging modules, and bandpass filters for wavelength-selective detection (Figure 1). To capture variability in imaging conditions, the device was operated free-hand in continuous video-capture mode at three nominal standoff distances (3, 6, and 12 inches) for each surface type and concentration level. A total of 41 video streams were recorded at approximately 16.2 frames per second (FPS), with a combined duration of 25.35 min. However, the 12-inch recordings were excluded from the final dataset because the increased distance reduced UV-C excitation intensity and limited the fluorescence response. Individual frames were subsequently extracted and manually selected based on image clarity, excluding those affected by UV-C LED saturation. Such saturation occurred primarily at shorter standoff distances when the imaging plane was nearly perpendicular to the LED illumination direction. Maintaining an angle of approximately 10–20° between the illumination direction and target surface helped minimize saturation. UV-C excitation induced autofluorescence from bacterial metabolites and cellular constituents, producing spatially localized fluorescence signals associated with bacterial concentration.

### 2.2. ROI Isolation and Background Removal

A total of 394 raw video frames were extracted after a manual quality check, and each inoculated sample was localized using fluorescence-intensity thresholding (Figure 1, isolation stage). A region of interest (ROI) was defined around each sample, and pixels outside the ROI were set to zero to remove extraneous fluorescence from the surrounding coupon surface and background. Each ROI was then cropped to a standardized spatial extent, yielding background-free, single-sample fluorescence images as input to the denoising stage.

### 2.3. Image Denoising: Pipeline Design and Selection

#### 2.3.1. Rationale for a Cascade Design

Raw UV-C fluorescence frames were affected by several co-occurring degradations. Low-photon acquisition introduced mixed Poisson–Gaussian noise, and the frames additionally exhibited uneven illumination, material-dependent autofluorescence background, and optical blur from the point-spread function (PSF). Because each classical restoration operator addresses only a specific noise or blur model, no single method could correct all of these degradations at once. Denoising was therefore implemented as an ordered cascade, in which the output of one operator was passed as the input to the next so that each stage acted on the residual degradation left by the preceding stage. Because these operators do not generally commute, both the selection and the ordering of operators were treated as explicit design variables and were evaluated systematically in the screening and cascade-formation steps described below.

#### 2.3.2. Candidate De-Noising Operators

Five candidate de-noising operators (DNOs) were evaluated: Wavelet (discrete wavelet transform, DWT) thresholding, NLM, unsharp masking, Wiener filtering, and RL-TV. Throughout the remainder of this manuscript, these de-noising operators are collectively denoted as DNOs. Wavelet thresholding decomposed each image into multiresolution sub-bands and shrank the high-frequency coefficients to suppress additive sensor noise while preserving edges. NLM reduced random noise by averaging across structurally similar image patches, thereby retaining fine spatial detail. Unsharp masking enhanced local contrast by subtracting a blurred copy of the image, increasing the visibility of fluorescence hotspots at the cost of amplifying high-frequency noise. Wiener filtering applied a frequency-domain minimum-mean-square-error criterion that balanced noise suppression against signal fidelity using the estimated signal-to-noise ratio. RL-TV iteratively inverted PSF blur under a Poisson forward model, with total-variation regularization added to constrain the ill-posed inversion and suppress noise amplification and ringing. Together, these operators spanned complementary restoration principles, which motivated evaluating them both individually and in combination.

#### 2.3.3. Operator Screening and Cascade Formation

Operator selection followed a two-stage screening strategy in which image-level fidelity assessment preceded classification-based evaluation [36,37]. In the first stage, each DNO was applied individually to every ROI and compared with the raw input using the signal-to-noise ratio (SNR), calculated as the ratio of raw-image signal power to the power of the difference between the raw and processed images and expressed in decibels. Under this definition, SNR measures fidelity to the raw image, with higher values indicating more conservative, structure-preserving processing and lower values indicating greater modification of the input. The SNR screening was used to identify the most fidelity-preserving operator for the cascade anchor and to characterize the relative behavior of the candidate operators before model training.

Cascades were then constructed from the DNOs retained after classification-based screening under two a priori ordering constraints. First, the DNO with the highest fidelity was fixed as the anchor so that the fluorescence signal was preserved before more aggressive processing. Second, because deconvolution can amplify noise as iterations proceed, noise-suppression stages were required to precede the deconvolution stage, restricting deconvolution to the final position. Because the DNOs do not commute, cascades were treated as ordered sequences. These constraints defined a limited set of candidate cascades that were selected systematically to evaluate the effects of operator combination and ordering.

In addition to the sequential cascades, a parallel multi-denoiser configuration was evaluated by stacking the outputs of the selected DNOs as separate input channels. The resulting multi-channel inputs were adapted to the pretrained backbone architecture while retaining the pretrained weights of the remaining layers. Unlike the sequential cascades, which apply operators successively, the parallel configuration presents the denoised representations simultaneously to the classifier. Candidate configurations were selected based on validation accuracy, with the best-performing model for each surface subsequently evaluated once on the held-out test set. All configurations used identical data splits.

### 2.4. Image Reconstruction and Dataset Preparation

Following denoising, each processed image (derived from a single raw fluorescence image) underwent spatial reconstruction to normalize fluorescence signal distribution. The reconstructed images were then partitioned to enrich spatial information for model training. Specifically, each image was divided into four equal, non-overlapping quadrants (top-left, top-right, bottom-left, and bottom-right). Deterministic rotations (0°, 15°, 30°, and 45°) were applied immediately after division, ensuring that each augmented sample retained at least two to three droplets and preserved meaningful fluorescence patterns. The quadrant images were subsequently combined with half-image and full-image representations to incorporate both local and global contextual information. All quadrant, half-image, and full-image representations were derived from the original fluorescence images and were therefore treated as augmented image samples rather than independent biological replicates. All images were then resized to 224 × 224 pixels to maintain a consistent input size across models and align with the default input resolution used during pre-training of the architectures (EfficientNet, ConvNeXt, YOLOv11-cls, and ViT), which were initialized with weights pre-trained on ImageNet.

To prevent data leakage, the training, validation, and testing partitions were defined at the level of raw fluorescence samples before any augmentation, so that no quadrant, half-image, full-image, or rotated derivative of a test sample appeared in the training or validation sets. To avoid class imbalance, the number of images per concentration class was balanced across splits. Additional augmentations, such as random rotations and horizontal and vertical flips, were applied only to the training and validation sets; the test set remained unaugmented to ensure a realistic evaluation. Four independent datasets were constructed, one per surface (Table 1).

### 2.5. Deep Learning Models, Training and Evaluation

#### 2.5.1. Architectures

Eight deep-learning classifiers spanning two architectural paradigms were benchmarked. The convolutional group comprised the YOLO11-cls family (n, s, m, l, and x variants), representing different model scales and providing a range of computational complexity and accuracy; EfficientNetB7, a compound-scaled convolutional network balancing network depth, width, and input resolution; and ConvNeXtBase, a modernized convolutional architecture incorporating depth-wise convolutions, inverted bottlenecks, and layer normalization. The attention-based group comprised a Vision Transformer (ViT), which partitions each image into fixed-size patches and uses multi-head self-attention to model global dependencies across the image. These architectures were selected to evaluate the effects of model capacity and different feature-learning approaches on fluorescence image classification. The five YOLO11-cls variants enabled comparison across model sizes within a common architecture family, while EfficientNetB7 and ConvNeXtBase provided complementary convolutional benchmarks. ViT was included to evaluate an attention-based alternative to convolutional feature extraction. All models were initialized with ImageNet-pretrained weights and fine-tuned on the fluorescence datasets. Including both paradigms enabled comparison of convolutional and attention-based feature representations for surface-dependent bacterial concentration classification.

#### 2.5.2. Training Configuration

All training and evaluation were performed on the University of Florida HiPerGator high-performance computing cluster. The YOLO11-cls models were trained using the Ultralytics framework, whereas EfficientNet, ConvNeXt, and ViT were implemented using Keras and PyTorch. All models were trained using the AdamW optimizer with an initial learning rate of 10^−4^, a cosine annealing learning-rate schedule, a batch size of 16, and cross-entropy loss. Training was conducted for up to 100 epochs with early stopping (patience = 10), and the checkpoint achieving the highest validation accuracy was selected for final evaluation. All eight architectures were subsequently compared based on their respective test-set accuracies. The highest-performing architecture for each surface was identified retrospectively to summarize comparative performance rather than to establish an independently validated optimal model. Training and evaluation were performed across four NVIDIA B200 GPUs, each with 80 GB HBM3e memory. The software environment included Ultralytics v8.4.110, PyTorch v2.11.0, and Python v3.12. The use of HiPerGator enabled efficient model training and analysis, reducing computational time during development. Once trained, the selected models can be deployed on appropriate computing hardware within scaled food-processing operations for automated contamination detection.

#### 2.5.3. Evaluation Metrics and Interpretability

Model performance was assessed on the held-out, unaugmented test set using overall accuracy together with per-class *Precision*, *Recall*, and *F1 score*, defined as Equations (1)–(4):

(1) $$\mathrm{Accuracy}=\;\frac{\mathrm{TP}+\mathrm{TN}}{\mathrm{TP}+\mathrm{TN}+\mathrm{FP}+\mathrm{FN}}$$

(2) $$\mathrm{Precision}=\frac{\mathrm{TP}}{\mathrm{TP}+\mathrm{FP}}$$

(3) $$\mathrm{Recall}=\frac{\mathrm{TP}}{\mathrm{TP}+\mathrm{FN}}$$

(4) $$F1\;\mathrm{score}=2\times \frac{P\times R}{P+R}$$

where *TP*, *TN*, *FP*, and *FN* denote true positives, true negatives, false positives, and false negatives, respectively. *Precision*, *Recall*, and *F*1 *score* were calculated for each class and reported as macro averages across the four concentration classes. Normalized confusion matrices were generated for the best-performing model on each surface to characterize classification errors across the four concentration classes.

To interpret the learned features, Eigen-CAM class-activation maps were generated for the trained models, enabling spatial identification of the discriminative fluorescence regions associated with each concentration level in the UV-C imagery.

## 3. Results

### 3.1. SNR-Based Denoising Operator Characterization

**Figure 2:**
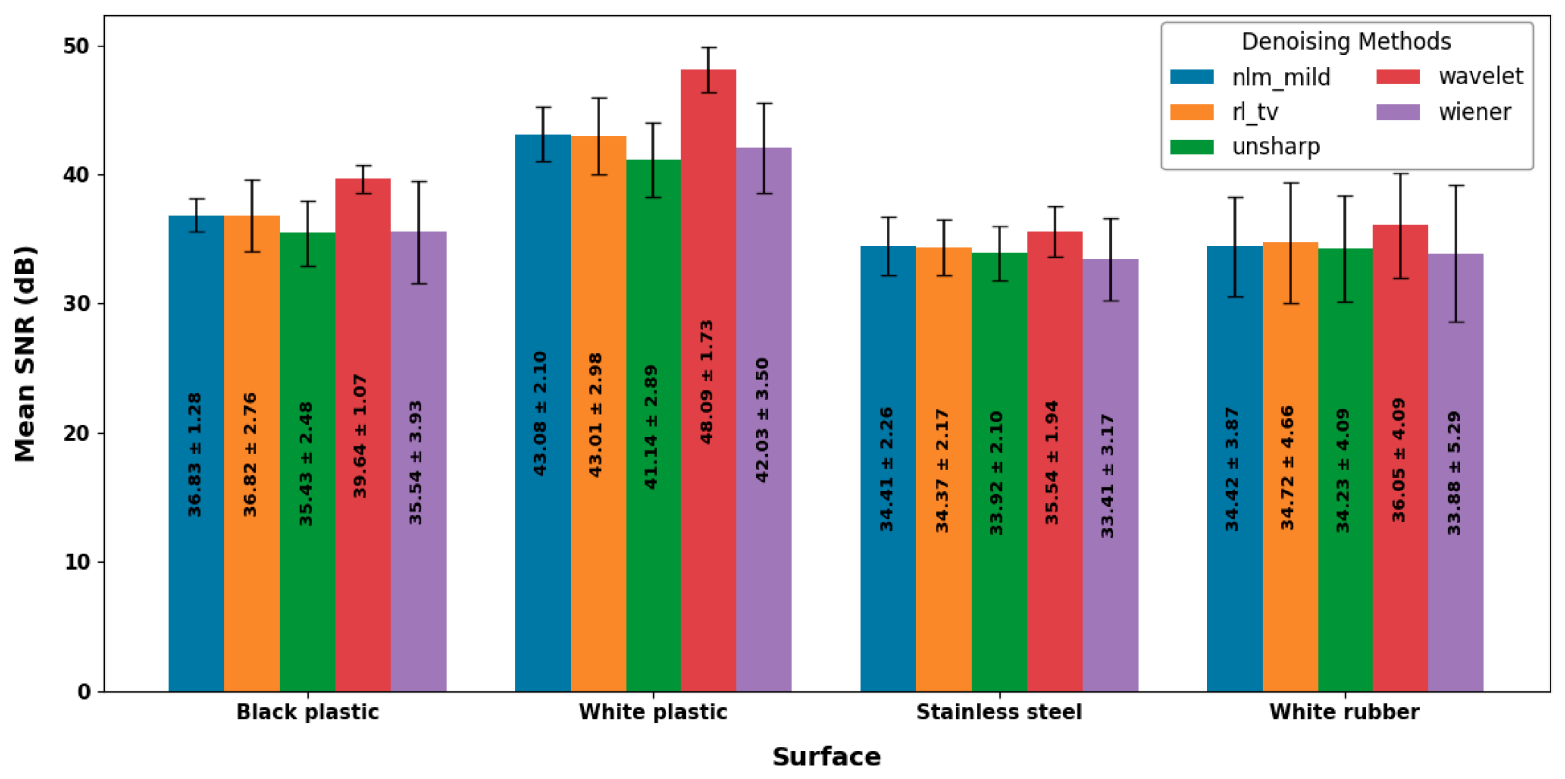
Mean signal-to-noise ratio (SNR, dB) ± standard deviation for five fluorescence image-denoising methods relative to the original raw images across four food-contact surfaces. The analysis included 76 original fluorescence frames for stainless steel, 194 for white rubber, 52 for white plastic, and 72 for black plastic. Lower SNR values indicate greater deviation from the original images, whereas higher values indicate greater similarity to the original images.

The five candidate DNOs were first evaluated individually against the raw fluorescence images using SNR (Figure 2). A higher SNR indicates greater fidelity to the raw input, whereas a lower SNR indicates greater image modification. This screening provided an initial assessment of operator behavior before model training and informed the selection of operators for subsequent cascade evaluation.

Wavelet thresholding produced the highest SNR on all four surfaces (40, 48, 36, and 36 dB for black plastic, white plastic, stainless steel, and white rubber, respectively), indicating the least modification of the raw images. Its consistently high SNR across surfaces supported its selection as the cascade anchor. The remaining operators exhibited a wider range of SNR values, with unsharp masking and Wiener filtering generally producing a lower SNR, whereas RL-TV and NLM showed intermediate values.

The SNR results provided an initial characterization of DNOs’ behavior but did not directly indicate classification performance. Greater image modification may reflect stronger suppression or enhancement and does not necessarily imply better or poorer classification, while DNOs with similar SNR characteristics may still provide different effects on the classification task. Therefore, individual-DNO classification performance was evaluated next to determine which operators were retained for subsequent cascade construction.

A surface-dependent pattern was also observed. White plastic exhibited the highest SNR range across all operators (41–48 dB), whereas the other surfaces showed lower ranges (33–40 dB), indicating that the evaluated operators produced less modification of fluorescence images from white plastic. This surface-dependent behavior motivated separate classification and cascade analyses for each surface rather than assuming a single universal preprocessing response.

### 3.2. Single-DNO Evaluation and Cascade Formation

**Figure 3:**
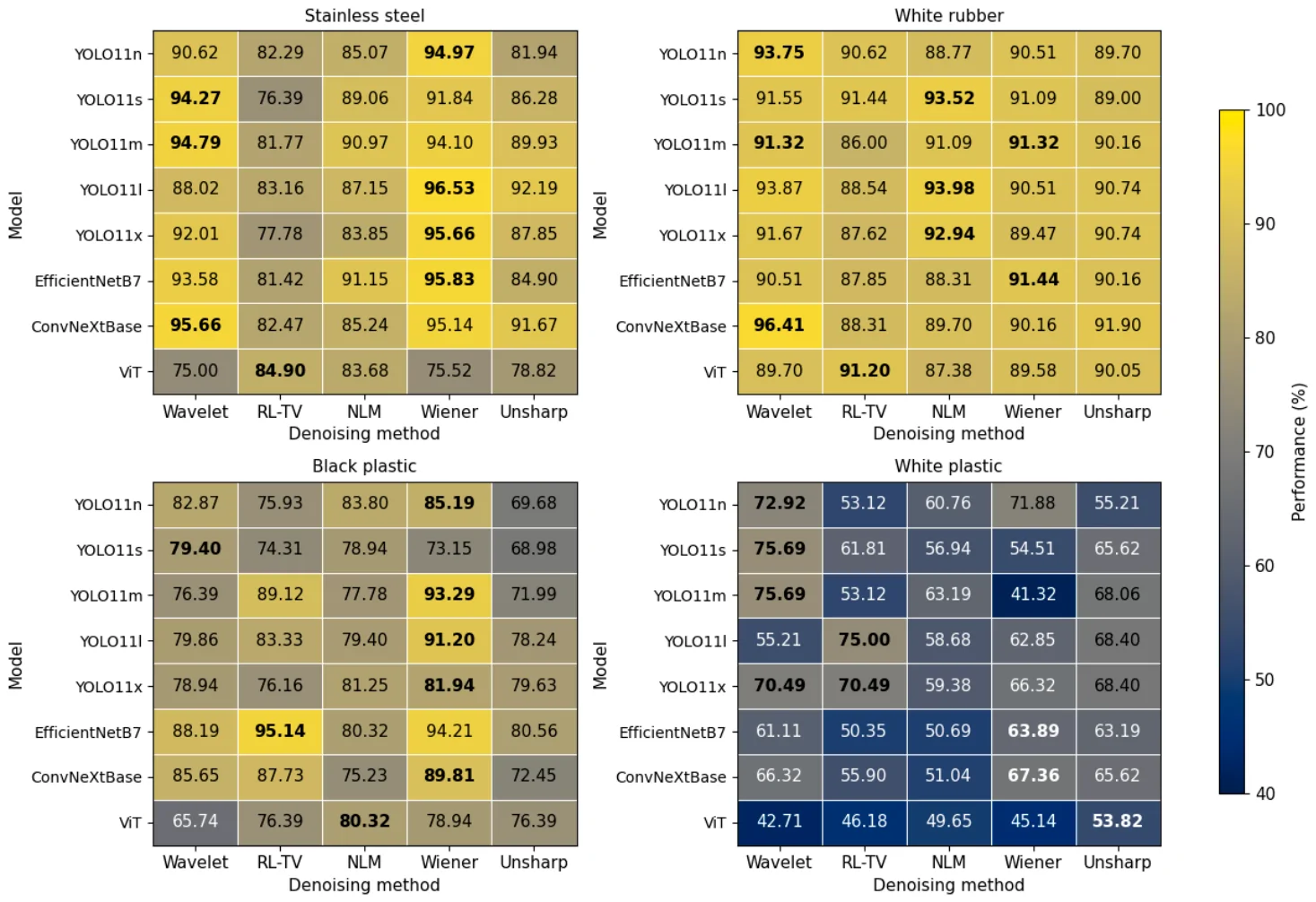
Effect of denoising methods on model classification performance by percent accuracy across different backgrounds: stainless steel, white rubber, black plastic, and white plastic.

**Figure 4:**
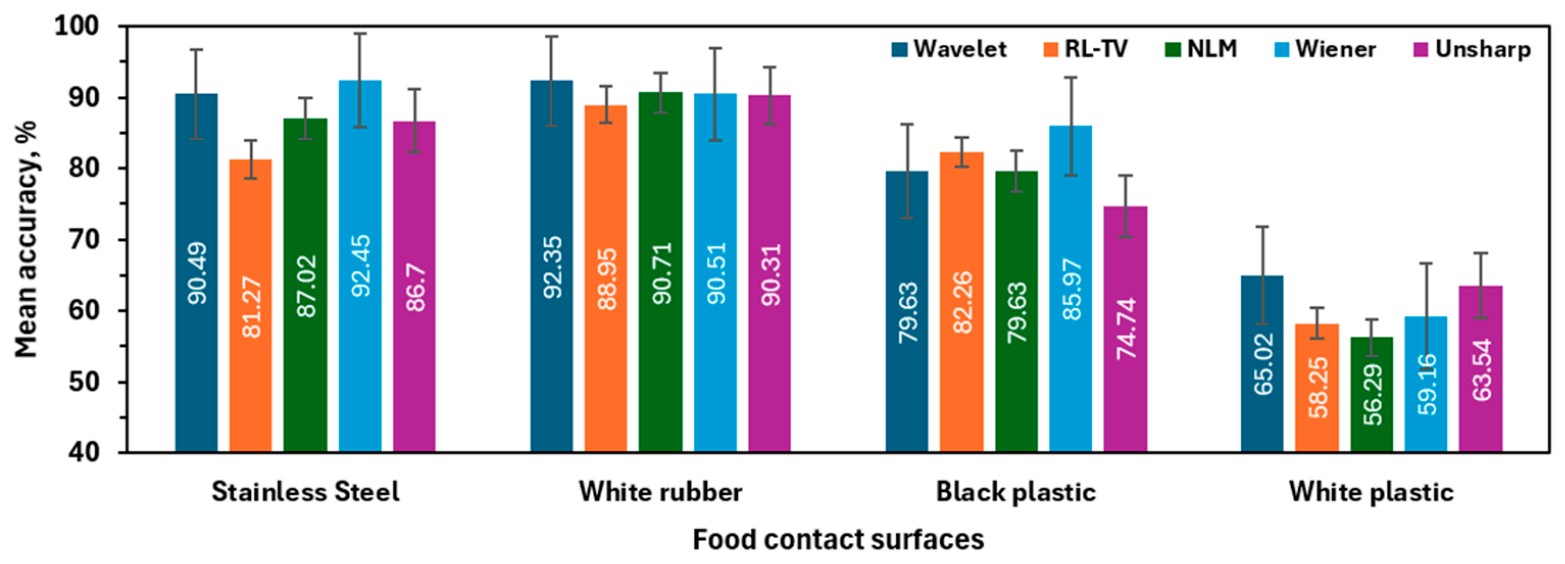
Mean classification accuracy of the evaluated models across the five denoising methods and four surfaces. Each bar represents the mean test accuracy across eight deep-learning architectures (*n* = 8), with error bars indicating ± standard deviation of the corresponding model accuracies.

Building on the SNR characterization, each DNO was evaluated based on classification performance across all eight models and four surfaces. Figure 3 presents the test accuracy for each model-DNO combination on each surface, establishing the single-DNO baseline for comparison with cascaded configurations and providing the empirical basis for selecting DNOs for combination. Figure 4 summarizes the mean performance of each model across the five DNOs and four surfaces, providing an overall assessment of model and DNO behavior. Together, these results revealed three performance patterns that guided the subsequent cascade design.

First, no single operator produced the highest accuracy across all surfaces. Considering the best-performing model for each surface, Wiener achieved the highest accuracy on stainless steel (96.53%), Wavelet on white rubber (96.41%) and white plastic (75.69%), and RL-TV on black plastic (95.14%). The observed surface-dependent performance suggests that the effectiveness of individual DNOs varies across imaging surfaces, emphasizing the need to evaluate preprocessing strategies across different materials rather than applying a single method uniformly.

Second, the results provided an empirical basis for pruning the candidate DNOs. NLM and unsharp masking had the lowest best-model mean accuracies across surfaces (83.01% and 83.32%, respectively), and neither achieved the highest accuracy on any surface. Their exclusion was therefore based primarily on their comparatively weak classification performance. RL-TV, despite its lower average performance, was retained because it achieved the highest accuracy on black plastic, where the other DNOs performed less effectively. Based on these results, Wavelet, Wiener, and RL-TV were selected as the cascade components. Wavelet was used as the anchor based on its highest SNR across surfaces and its strong single-DNO classification performance, achieving the highest accuracy on two surfaces and competitive performance on a third.

Third, convolutional models generally outperformed the attention-based ViT across the evaluated model–DNO combinations. ViT was the weakest model on most combinations and showed particularly low accuracy on white plastic, ranging from 42.71% to 53.82% across DNOs. The mean-performance results in Figure 4 further illustrate this overall difference between the convolutional and transformer-based architectures. These results indicate that the convolutional architectures provided more effective classification performance for the fluorescence data under the conditions evaluated.

Guided by these findings, sequential cascades were constructed from wavelet, Wiener, and RL-TV under the two a priori ordering constraints established previously, with wavelet anchored first and RL-TV confined to the final stage because of its potential to amplify noise. Because the DNOs do not commute, cascade order was treated as an explicit design factor. A predefined set of candidate cascades was therefore evaluated to examine the effects of combining denoisers, cascade order, and the inclusion and position of the deconvolution stage, as summarized in Table 2. These candidate configurations were designed not only to compare cascade performance but also to explicitly test the proposed ordering rationale, including the use of Wavelet as the anchor and the placement of RL-TV after noise-suppression stages. The final cascade order was therefore determined from these physically motivated constraints together with the empirical comparison of alternative operator sequences. In addition, a parallel configuration combining all three denoisers through multi-channel stacking was evaluated to compare sequential and parallel strategies for exploiting complementary denoising information.

### 3.3. Cascade Comparison and Selection of the Best Pipeline

The candidate cascades and parallel multi-channel configuration were trained and evaluated using the same models, training protocol, and data splits as the single-DNO baselines. For each surface, the best-performing model was used to ensure direct comparison across configurations. Table 3 reports test accuracy and the corresponding best-performing model for each surface, with the best single-DNO result serving as the reference baseline.

The three-stage cascade Wavelet → Wiener → RL-TV achieved the highest mean accuracy (94.18%) and was the best-performing or close to best method on stainless steel, white rubber, and white plastic, making it the strongest overall pipeline. On stainless steel, its accuracy (97.57%, YOLO11 s) was only 0.17 percentage points below the highest observed accuracy (97.74%, Wavelet → Wiener with EfficientNetB7). On white rubber (97.57%, YOLO11 m) and white plastic (87.15%, YOLO11 s), it achieved the highest accuracy among the evaluated configurations. Its largest improvement occurred on white plastic, where it exceeded the best single DNO result by 11.46 percentage points (75.69%) and the parallel configuration by 10.41 percentage points (76.74%). Notably, the surface that showed the least modification during the initial denoising screening benefited most from the full cascade. These results suggest that sequentially combining complementary denoising and deconvolution operations can improve classification performance beyond that achieved by individual DNOs, particularly for the more challenging white plastic surface. Overall, the three-stage cascade provided a consistent high-performing preprocessing strategy across the evaluated surfaces, reducing the surface dependence observed with individual operators.

Among the three DNO cascades containing the same DNOs, placing deconvolution last produced substantially higher mean accuracy (94.18%) than placing it in the middle (Wavelet → RL-TV → Wiener, 87.24%). The ordering-control cascade with deconvolution first (RL-TV → Wavelet → Wiener) also performed poorly on white plastic (68.75%) while maintaining higher accuracy on the other surfaces. Together, these results support placing noise-suppression operations before deconvolution in the evaluated configurations. The deconvolution stage also provided an additional benefit, as the three-stage Wavelet → Wiener → RL-TV cascade exceeded the two-stage Wavelet → Wiener cascade (91.48%), which in turn exceeded the best single-DNO mean accuracy (90.94%). The Wiener-anchored variant (Wiener → Wavelet → RL-TV, 87.88%) performed substantially below the corresponding wavelet-anchored cascade, supporting the use of wavelet as the anchor. In contrast, the two DNO orderings produced similar mean accuracies (91.48% versus 90.70%), indicating that order had a smaller effect when only the two DNOs were combined.

The sequential and parallel multi-denoiser strategies also showed different performance patterns. The sequential cascade outperformed the parallel multi-channel configuration on all four surfaces, with the largest difference observed on white plastic (87.15% versus 76.74%). Mean accuracy was also higher for the sequential cascade (94.18%) than for the parallel configuration (90.94%). The parallel configuration matched the best single-DNO mean accuracy (90.94%), whereas the sequential cascade provided a 3.24-percentage-point improvement. These results indicate that explicitly ordering complementary preprocessing operations was more effective than presenting their outputs simultaneously as separate channels under the conditions evaluated. The advantage was particularly noticeable on white plastic, where the initial denoising analysis indicated greater residual background signal. Thus, the manner in which complementary denoisers are combined can substantially influence classification performance, with sequential fusion providing the stronger overall strategy in this study.

One exception was observed on black plastic, where the single RL-TV DNO achieved 95.14% accuracy with EfficientNetB7, compared with 94.44% for the best cascade using YOLO11 x, a difference of 0.70 percentage points. Although the cascade did not achieve the highest accuracy on this surface, it remained within one percentage point of the best-performing configuration while achieving the highest accuracy on the other three surfaces. This consistency across surfaces distinguishes the Wavelet → Wiener → RL-TV cascade from the individual DNOs and other evaluated configurations and supports its selection as the most robust overall preprocessing strategy.

Although the cascade generalized across surfaces at the preprocessing level, model performance remained surface-dependent, suggesting two possible deployment strategies. No single architecture achieved the highest accuracy on all surfaces. With the selected Wavelet → Wiener → RL-TV cascade, the highest per-surface accuracy was achieved by YOLO11 s on stainless steel (97.57%) and white plastic (87.15%), YOLO11 m on white rubber (97.57%), and YOLO11 x on black plastic (94.44%). For a general-purpose system using a single fixed model across all surfaces, the Wavelet → Wiener → RL-TV cascade paired with YOLO11 L provides a consistent option, achieving accuracies of 97.05%, 97.11%, 92.13%, and 85.76% on stainless steel, white rubber, black plastic, and white plastic, respectively, with a mean accuracy of 93.00%. In contrast, selecting the best-performing model for each surface yielded a mean accuracy of 94.18%, suggesting that surface-specific model selection may offer improved performance.

### 3.4. Error Mode Analysis

**Figure 5:**
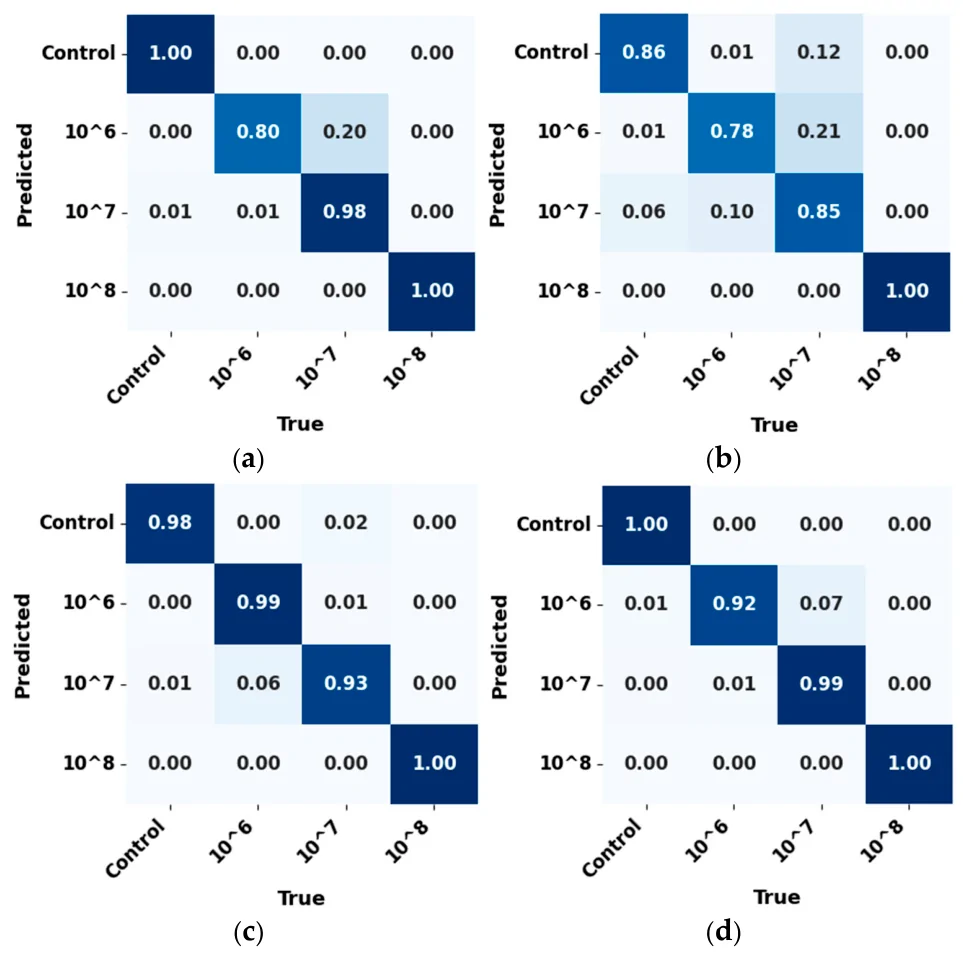
Normalized confusion matrices for the best-performing models under different input configurations: (**a**) Black plastic, (**b**) white plastic, (**c**) stainless steel, and (**d**) white rubber. The corresponding test accuracies were 94.44% for black plastic (YOLO11 x-cls), 87.15% for white plastic (YOLO11 s-cls), 97.57% for stainless steel (YOLO11 s-cls), and 97.57% for white rubber (YOLO11 m-cls).

To gain deeper insight into the classification behavior of the best-performing models on each surface, normalized confusion matrices were analyzed (Figure 5). The matrices show the proportion of correct predictions along the diagonal and the patterns of misclassification across the four concentration classes (Control, 10^6^, 10^7^, and 10^8^ CFU/mL).

On black plastic (Figure 5a), the model achieved 94.44% accuracy, with the Control and 10^8^ CFU/mL classes classified perfectly. The dominant error involved the 10^6^ CFU/mL class, for which 20.37% of samples were classified as 10^7^ CFU/mL. In contrast, 98.15% of true 10^7^ samples were correctly classified, with only 0.93% assigned to Control and 0.93% to 10^6^ CFU/mL. Thus, the primary error mode on black plastic was the upward classification of the lower bacterial concentration as the next higher concentration rather than confusion between contaminated and Control samples.

White plastic (Figure 5b) showed the lowest overall accuracy (87.15%) and the most pronounced misclassification among the evaluated surfaces. Although the 10^8^ CFU/mL class was classified perfectly, greater confusion occurred among the Control, 10^6^, and 10^7^ CFU/mL classes. Specifically, 12.50% of Control samples (30 of 240 test images) were misclassified as 10^7^ CFU/mL, while 20.83% of true 10^6^ samples were classified as 10^7^ CFU/mL. The misclassification of Control samples may lead to false alarms in practical screening applications, although the 12.50% proportion does not represent the overall false-positive rate. Conversely, 9.72% of true 10^7^ samples were classified as 10^6^ CFU/mL and 5.56% as Control. These results highlight the challenges associated with white plastic, particularly in distinguishing uncontaminated samples from lower contamination levels and discriminating between adjacent bacterial concentrations.

On stainless steel (Figure 5c), classification was highly accurate overall (97.57%). The 10^8^ CFU/mL class was classified perfectly, and 99.31% of true 10^6^ samples were correctly identified. The largest error occurred for the 10^7^ CFU/mL class, with 6.25% of samples classified as 10^6^ CFU/mL and 0.69% as Control. The Control class was also classified reliably, with 97.92% of samples correctly identified. Thus, errors on stainless steel were concentrated primarily between the two intermediate bacterial concentrations.

White rubber (Figure 5d) also achieved 97.57% accuracy and showed relatively little confusion across classes. The Control and 10^8^ CFU/mL classes were classified perfectly, while the primary error again occurred between 10^6^ and 10^7^ CFU/mL. Specifically, 7.41% of true 10^6^ samples were classified as 10^7^ CFU/mL, whereas only 0.93% of true 10^7^ samples were classified as 10^6^ CFU/mL. The 10^7^ class was otherwise correctly identified in 98.61% of cases.

Across all four surfaces, the dominant error mode involved confusion between the lower bacterial concentrations, particularly the upward misclassification of 10^6^ as 10^7^ CFU/mL, whereas the highest concentration (10^8^ CFU/mL) was classified perfectly on every surface. Misclassifications were therefore predominantly between neighboring concentration classes rather than distant classes, indicating that the models generally captured the concentration gradient. Confusion between Control and contaminated samples was limited on black plastic, stainless steel, and white rubber but was more pronounced on white plastic, which retained the greatest residual ambiguity despite the substantial improvement provided by the cascade. Notably, the Wavelet →Wiener → RL-TV cascade increased white-plastic accuracy to 87.15%, compared with 75.69% for the best single operator, indicating that sequential preprocessing substantially mitigated the surface-specific limitations observed with individual denoising methods. Overall, the error patterns suggest that the cascade effectively improved separation of contaminated and Control samples while the remaining challenge was primarily discrimination between closely spaced lower bacterial concentrations. To further evaluate classification performance beyond overall accuracy, macro-averaged precision, recall, and F1-score were calculated for the best-performing surface-specific models (Table 4). These metrics provide a more comprehensive assessment of classification performance by assigning equal weight to all four concentration classes.

### 3.5. Explainability Analysis

**Figure 6:**
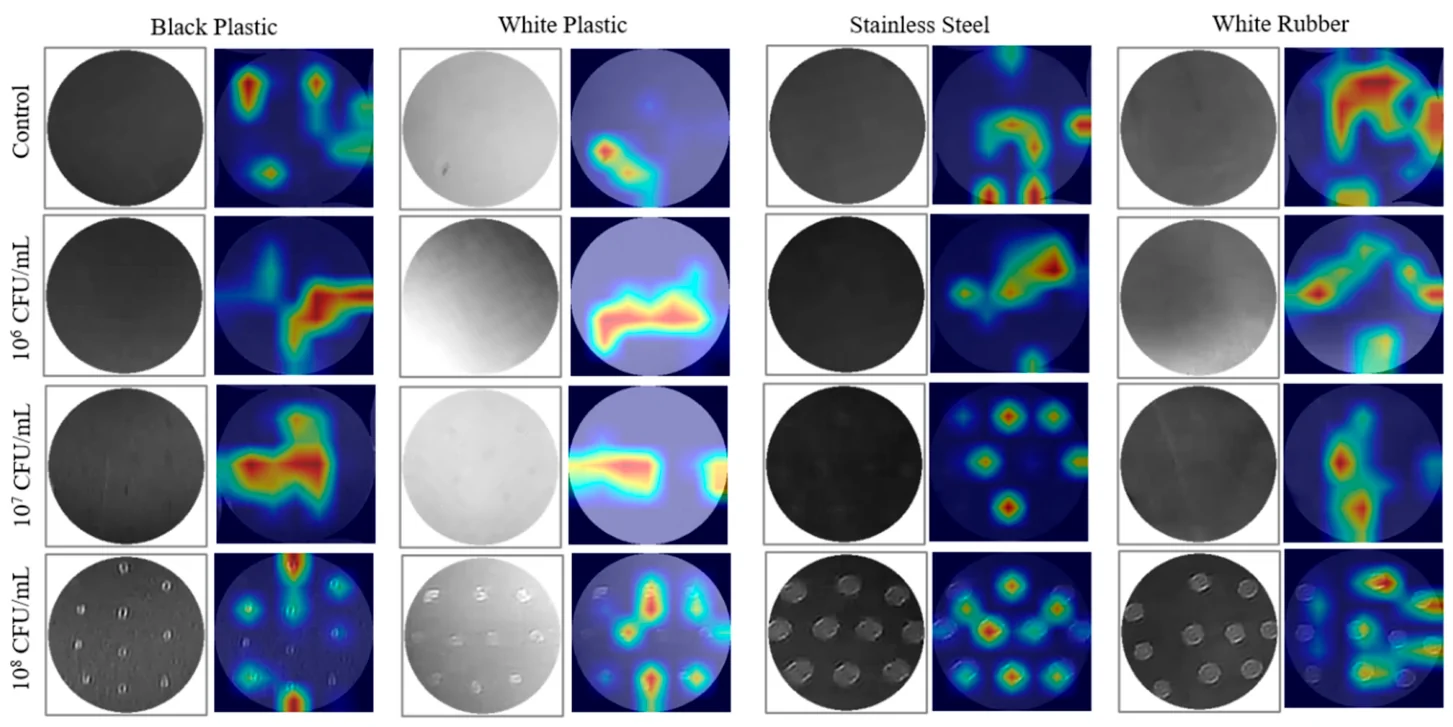
Denoised fluorescence images and corresponding Eigen-CAM heat maps illustrating discriminative regions identified by the best-performing deep-learning models across four food-contact surfaces and *E. coli* concentration levels. Warmer colors indicate stronger relative model activation within each image. Heat maps were independently normalized for visualization; therefore, activation intensities should not be compared quantitatively across images.

Figure 6 shows the denoised fluorescence images and corresponding Eigen-CAM heat maps for the best-performing pipeline across the four surfaces and *E. coli* concentration levels. The heat maps illustrate the image regions that contributed most strongly to the model’s classification decisions. As concentration increased, the activation patterns generally became stronger and more spatially concentrated, with higher-concentration images showing more localized regions of activation, particularly on stainless steel and white rubber. These patterns qualitatively coincide with the strong classification performance observed at the highest concentration on these surfaces. In contrast, activation on white plastic remained comparatively diffuse across concentration levels, with responses extending beyond localized regions. This behavior is consistent with the greater residual background observed during SNR analysis and the remaining classification errors on white plastic. However, the Eigen-CAM maps should be interpreted cautiously, as activated regions represent features that the model considered discriminative for classification and do not necessarily correspond directly to *E. coli* fluorescence, individual droplets, or imaging artifacts. Thus, the Eigen-CAM results provide qualitative insight into the spatial features used by the model but do not, by themselves, establish the physical origin of those features.

## 4. Discussion

This study demonstrates that preprocessing effectiveness in UV-C fluorescence classification depends not only on the choice of individual DNO but also on the complementary effects achieved through DNO combinations. The superior performance of sequential processing over parallel multi-channel fusion suggests that explicitly ordered preprocessing is advantageous when fluorescence images contain multiple, co-occurring degradations. The ordering experiments further showed that the position of the deconvolution stage was particularly important. Placing RL-TV deconvolution after noise-suppression stages produced substantially better performance than placing it earlier, consistent with the sensitivity of Richardson–Lucy deconvolution to noise amplification [23]. In contrast, reversing the order of the two DNOs produced a relatively small change in performance, suggesting that the major ordering constraint arises from the interaction between noise suppression and deconvolution rather than from denoising order alone.

The surface-dependent results provide further insight into the role of preprocessing. Individual DNOs showed different levels of effectiveness across materials, indicating that preprocessing performance was influenced by surface-dependent fluorescence and background characteristics. White plastic was particularly challenging for the individual DNOs but also benefited most from the cascaded approach. This suggests that combining complementary operations can mitigate residual degradations that are not adequately addressed by any single method. Nevertheless, surface effects were not completely eliminated. Although the selected cascade provided a common preprocessing strategy across the four materials, the most effective classifier still varied by surface, indicating that residual differences in image characteristics remained relevant to feature representation.

The model comparison further supports the suitability of convolutional architectures for this application. The YOLO11 models generally outperformed ViT across the evaluated conditions, particularly on the more challenging white-plastic surface. This may reflect the localized nature of bacterial fluorescence patterns and the limited size of the task-specific dataset, for which the local inductive bias of convolutional architectures can be advantageous relative to global self-attention [38,39]. The results therefore favor convolutional architectures for the present application, while the weaker performance of ViT suggests that transformer-based approaches may require larger domain-specific datasets or hybrid architectures to realize their potential.

The error analysis provides an additional perspective on the practical significance of the results. Most misclassifications occurred between neighboring bacterial concentration levels rather than between contaminated and Control samples. The models therefore showed greater difficulty in resolving relatively similar contamination levels than in distinguishing contaminated samples from clean controls. This distinction is important for screening applications, where reliable identification of contamination is more consequential than precise discrimination between adjacent contamination levels. The remaining errors on white plastic, particularly those involving the Control and lower concentration classes, indicate that surface-dependent background effects remain an important consideration even after cascaded preprocessing.

Previous CSI-D+ studies investigated *E. coli* concentration classification under different experimental conditions using a 1st Generation SafetySpect CSI-D+, including citrus and spinach leaves with eight concentration levels [35] and stainless steel and white rubber with four concentration levels [4]. In contrast, the present study extends these investigations using a 2nd Generation CSI-D+ by systematically evaluating five denoising operators, alternative cascade configurations, and eight deep-learning classifiers across four food-contact surfaces. However, differences in 2nd Gen image resolution and LED source design, dataset construction, and evaluation protocols limit direct comparisons of reported classification accuracies.

From a deployment perspective, the results suggest a trade-off between simplicity and surface-specific optimization. A fixed model paired with the selected cascade provides a single configuration without requiring prior surface identification, whereas adapting the classifier to the detected material offers additional performance potential. However, the second option requires an independent surface-identification mechanism and validation-based model selection before it can be implemented as a deployable strategy.

Three limitations are particularly relevant to practical application. First, the surface-aware performance represents a retrospective benchmark because the best-performing model for each material was identified using test-set results. Although a fixed random seed (42) was used to ensure consistent experimental settings, future studies should incorporate repeated-seed evaluations and statistical uncertainty analyses to further assess the robustness of the observed performance differences. Second, the experiments were conducted under controlled conditions using predefined concentration levels of a single *E. coli* strain. Therefore, the results demonstrate concentration-level classification rather than species-specific identification in unknown microbial mixtures. Further validation involving other microorganisms, organic residues, and lower contamination levels is necessary to establish detection specificity. Third, the findings may not directly generalize to untested surfaces such as glass, wood, or ceramic, as differences in surface reflectance, intrinsic fluorescence, absorption, roughness, and porosity may affect fluorescence responses and classification performance. Future validation should therefore consider a broader range of surface materials, imaging geometries, including standoff distance, and contamination scenarios.

## 5. Conclusions

This study developed and evaluated an AI-based framework for classifying *E. coli* cell concentration on four optically distinct food-contact surfaces using UV-C fluorescence imaging from the CSI-D+ handheld device, a systematically designed denoising pipeline, and eight deep-learning classifiers. The results demonstrate that, for fluorescence images affected by multiple co-occurring degradations, how complementary DNOs are combined can be as important as the choice of individual DNOs. An ordered Wavelet → Wiener → RL-TV cascade emerged as the best-performing pipeline, achieving a mean test accuracy of 94.18% across the four surfaces. The cascade outperformed both the strongest single-DNO configuration and the parallel multi-channel configuration on every surface, with the largest improvement observed on the challenging white-plastic surface. The superior performance of the sequential, physically motivated cascade and the ordering experiments support placing noise-suppression operations before regularized deconvolution. This finding highlights the importance of processing order when combining preprocessing DNOs.

The framework also clarified how surface variability influences classification performance. The selected cascade provided a common preprocessing strategy across surfaces, although the optimal model remained surface-dependent, with no single architecture achieving the highest accuracy on all materials. The convolutional YOLO models generally outperformed ViT. For practical use, the cascade paired with YOLO11 L-cls provided a general-purpose configuration with consistent performance across the evaluated surfaces. Error analysis further revealed that most misclassifications occurred between adjacent concentration levels rather than between contaminated and Control samples. This finding indicates that the primary remaining challenge was distinguishing between closely spaced contamination levels rather than detecting contamination itself, provided that the contamination level was sufficiently high to produce detectable fluorescence (≥10^6^ CFU/mL).

Although the proposed framework demonstrated promising classification performance on a high-performance supercomputer, its computational efficiency under practical deployment conditions remains to be evaluated on a handheld AI-enabled embedded controller/processor. Future work will assess preprocessing latency, model inference time, and end-to-end computational performance on target deployment hardware, along with validation across additional surface materials, contamination levels, and application scenarios. These efforts could advance UV-C fluorescence imaging with ordered denoising cascades and compact convolutional models toward rapid, non-destructive, on-site monitoring of various bacterial contamination on diverse food-contact surfaces.

## Figures and Tables

**Table 1:** Distribution of original raw fluorescence frames and derived quadrant (Q), half-frame (H), and full-frame (F) images used for training, validation, and testing across four food-contact surfaces and *E. coli* concentration classes. Values in parentheses indicate the number of frames or images per concentration class.

Dataset	Surface	Classes	Raw Frames (per Class)	Train (per Class)	Val (per Class)	Test (per Class)
Set 1	Stainless steel	4	76 (14–21)	18,720 (4680)	1920 (480)	1280 (320)
Set 2	White rubber	4	194 (32–76)	50,400 (12,600)	5760 (1440)	1920 (480)
Set 3	White plastic	4	52 (9–21)	18,720 (4680)	1920 (480)	960 (240)
Set 4	Black plastic	4	72 (12–22)	18,720 (4680)	1920 (480)	960 (240)

Raw frames represent the original fluorescence frames manually extracted from video streams after quality screening. Training, validation, and test counts represent the derived Q, H, and F image representations rather than independent raw frames.

**Table 2:** Candidate denoising cascades and the design question addressed by each configuration.

Cascade	Design Question
Wavelet → Wiener	Does combining two denoisers help, and does order matter?
Wiener → Wavelet	
Wavelet → Wiener → RL-TV	Does the deconvolution stage help, and in which position?
Wavelet → RL-TV → Wiener	
Wiener → Wavelet → RL-TV	Must Wavelet be the anchor?
RL-TV → Wavelet → Wiener	Is denoising-before-deconvolution justified?

**Table 3:** Test accuracy (%) of the single-DNO baseline, sequential cascades, and parallel multi-channel configuration, with the best-performing model for each surface shown in parentheses.

Method	Stainless Steel	White Rubber	Black Plastic	White Plastic	Mean
Best single operator	96.53 (YOLO11 L)	96.41 (ConvNeXtBase)	95.14 (EfficientNetB7)	75.69 (YOLO11 s)	90.94
Wavelet → Wiener	97.74 (EfficientNetB7)	96.06 (YOLO11 L)	91.90 (YOLO11 L)	80.21 (YOLO11 L)	91.48
Wiener → Wavelet	96.35 (YOLO11x)	94.68 (YOLO11l)	90.51 (EfficientNetB7)	81.25 (YOLO11 s)	90.70
Wavelet → RL-TV → Wiener	94.10 (YOLO11 m)	96.41 (YOLO11 L)	86.57 (ConvNeXtBase)	71.88 (YOLO11 n)	87.24
Wiener →Wavelet → RL-TV	92.01 (YOLO11 L)	94.79 (YOLO11 m)	87.96 (EfficientNetB7)	76.74 (YOLO11 L)	87.88
RL-TV → Wavelet → Wiener	95.31 (YOLO11 m)	97.11 (YOLO11 s)	93.06 (EfficientNetB7)	68.75 (YOLO11 L)	88.56
Wavelet → Wiener → RL-TV	97.57 (YOLO11 s)	97.57 (YOLO11 m)	94.44 (YOLO11 x)	87.15 (YOLO11 s)	94.18
Parallel (multi-channel stacking)	97.22 (ConvNeXtBase)	95.83 (YOLO11s)	93.98 (EfficientNetB7)	76.74 (YOLO11 L)	90.94

**Table 4:** Classification accuracy and macro-averaged precision, recall, and F1-score of the best-performing surface-specific deep-learning models using the sequential Wavelet → Wiener → RL-TV denoising cascade across four food-contact surfaces.

Surface	Final Model	Test Accuracy (%)	Macro-P (%)	Macro-R (%)	Macro-F1 (%)
Stainless steel	YOLO11s-cls	97.57	97.50	97.54	97.49
White rubber	YOLO11m-cls	97.57	97.75	97.83	97.74
Black plastic	YOLO11x-cls	94.44	94.50	95.21	94.45
White plastic	YOLO11s-cls	87.15	87.26	88.04	87.44

## Data Availability

The image datasets presented in this article are not readily available due to proprietary restrictions of the sponsoring company. Requests to access the datasets should be directed to Thomas F. Burks (tburks@ufl.edu).

## Abbreviations

The following abbreviations are used in this manuscript:

CSI-D^+^	Contamination and Sanitization Inspection and Disinfection Plus
UV-C	Ultraviolet C radiation
CFU	Colony-forming unit
NLM	Non-local means
RL-TV	Richardson–Lucy deconvolution with total-variation regularization
YOLO	You only look once
ViT	Vision transformer
DNO	Denoising operator
SNR	Signal to noise ratio
